# Quantifying Area Back Scatter of Marine Organisms in the Arctic Ocean by Machine Learning-Based Post-Processing of Volume Back Scatter

**DOI:** 10.3390/s25103121

**Published:** 2025-05-15

**Authors:** Ole Arve Misund, Anna Nikolopoulos, Vegard Stürzinger, Haakon Hop, Paul Dodd, Rolf J. Korneliussen

**Affiliations:** 1 Norwegian Polar Institute, Fram Centre, 9296 Tromsø, Norway; anna.nikolopoulos@npolar.no (A.N.); vegard.stuerzinger@gmail.com (V.S.); haakon.hop@npolar.no (H.H.); paul.dodd@npolar.no (P.D.); 2 Institute of Marine Research, 5005 Bergen, Norway; rolf.korneliussen@hi.no; 3 UiT The Arctic University of Norway, 9019 Tromsø, Norway

**Keywords:** acoustic backscatter, LSSS, zooplankton classification, icebreaking noise removal, post-processing, Central Arctic Ocean

## Abstract

As the sea ice reduces in both extent and thickness and the Arctic Ocean opens, there is substantial interest in mapping the marine ecosystem in this remote and until now largely inaccessible ocean. We used the *R/V Kronprins Haakon* during surveys in the Central Arctic Ocean (CAO) in 2022 and 2023 to record the marine ecosystem using modern fisheries acoustics and net sampling. The 2022 survey reached all the way to the North Pole. In a first, principally manually based post-processing of these acoustic recordings using the Large-Scale Survey Post-processing System (LSSS), much effort was used to remove segments of noise due to icebreaking operations. In a second, more sophisticated post-processing, the KORONA module of LSSS with elements of machine learning was applied for further noise reduction and to allocate the area back-scattering recordings to taxonomic groups as order, families and even species of fish and plankton organisms. These results highlight the need for further advances in post-processing systems to enable the direct allocation of back-scattered acoustic energy to taxonomic categories, including species-level classifications.

## 1. Introduction

In open waters worldwide, marine ecosystems are surveyed using advanced fisheries acoustics [1]. By applying this method, the distribution of commercially important fish and plankton species can be mapped and their biomass estimated [2,3,4]. The method, instruments, protocols, and platforms for marine ecosystem surveys, using fisheries acoustics, have been much improved since the first attempts in the early sixties [5]. The method has been proven to be linear meaning that the acoustic back-scattering strength increases proportionally with the density of scattering organisms [6], the target strengths for important species have been measured, the instruments have produced reliable digital outputs [7,8], the protocols including calibration have been standardized, and the low-noise research vessels with the transducers mounted on a protruding keel have been evaluated as efficient platforms [9].

Since the nineties, digital post-processing systems have been developed to help allocate recordings to specific species [10,11,12]. Using such systems, noise and unwanted signals can be deleted, specific settings regulated, and layers set conveniently. Even the target strength of single organisms identified can be measured underway. Some of these systems now have elements of machine learning to help the scrutinizing process by removing noise automatically and suggesting the allocation of back scatter to given species, families, or other taxonomic units of marine organisms.

Here, we describe the application of the LSSS post-processing system [13] using its KORONA module [14] with elements of machine learning to survey the European sector of the Arctic Ocean. By using the LSSS, a nearly continuous acoustic mapping of the upper 500 m of the water column was obtained by removing the segments cluttered with substantial noise from icebreaking. Finally, perspectives for the further development of the post-processing system for better and more efficient mapping and monitoring of marine ecosystems are discussed.

## 2. Materials and Methods

Acoustic data from two cruises with *R/V Kronprins Haakon* (KPH) in the Arctic Ocean in 2022 and 2023 were collected for this study. The surveys started in the productive open waters near Svalbard and were run all the way to the ice-covered waters in the Central Arctic Ocean (CAO). The first survey (2022) was conducted all the way to the North Pole. KPH is a modern Polar Class 3, 11,000 GRT icebreaking research vessel [15]. KPH is equipped with state-of-the-art instruments for broadband acoustic surveying [16] of the water column along the ship track. Continuous recordings were made by the hull-mounted transducers of the Simrad EK80 echosounder, operated at 18, 38, 70, 120, 200, and 333 kHz. The beam width (−3 dB points) was 11° on the 18 kHz transducer operated at 1.6 kW. For the other frequencies, the beam width was 7°, and the transducers operated from 2.0 kW for 38 kHz to 0.05 kW for the 333 kHz transducer. All frequencies operated with continuous wave (CW) pulses and fast ramping. The volume back-scattering coefficient (s_v_) at the six frequencies was recorded and stored from surface to 500 m depth.

**Figure 1:**
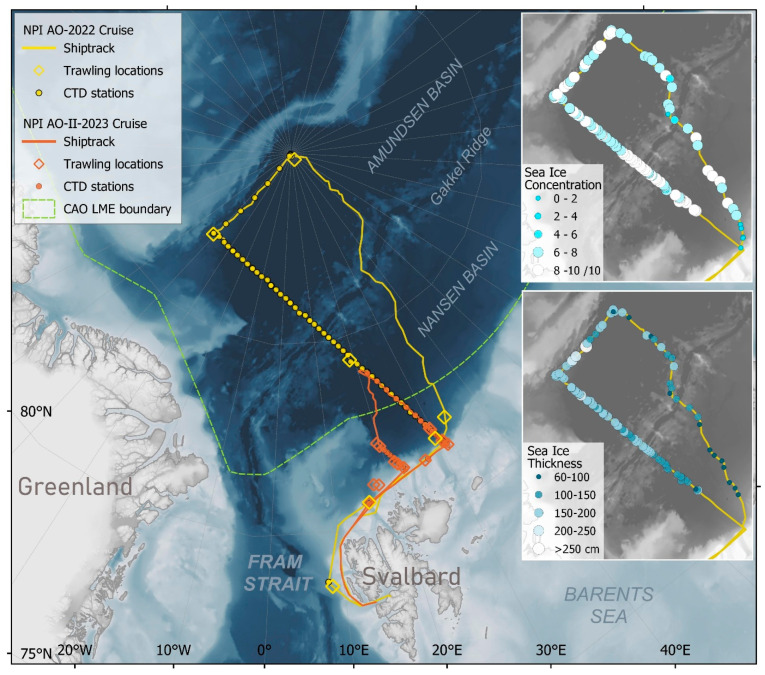
Bathymetric map of the Eurasian sector of the Arctic Ocean overlaid by the ship track, CTD stations, and trawling locations of the two Norwegian Polar Institute (NPI) cruises in yellow (2022) and red (2023). The upper right insert figure shows the sea ice concentration (observed on a scale from 1–10) and the lower right insert figure shows the thickness of sea ice. These underway ship-based sea ice observations were performed according to the Arctic Shipborne Sea Ice Standardization Tool (ASSIST) ice-watch protocol during the 2022 cruise.

Both cruises started from Longyearbyen, Svalbard. The first cruise [17] started on 22 July 2022, and data collection was initiated when the ship departed the harbour (Figure 1). The ship followed a route west of Svalbard, north on the Svalbard shelf, encountering the sea ice at 82°05′ N, and then sailed on a northern course all the way to the North Pole. From there, the ship continued a southwestern route along 41° W to 87°10′ N before turning southeast along an established hydrographic transect, started in 2021 [18]. Data collection was stopped on 19 August 2022, at 81°31′ N 31°06′ E at ship log 10,175. The second, shorter cruise [19] was conducted in the period 12–27 August 2023 and focused in more detail on the ecosystem on the shelf slope northeast of Svalbard and into the deeper Nansen Basin (Figure 1). That year, the ice edge was encountered at 81°32.7′ N 22°10′ E. The ship manoeuvred quite easily through the approximately 1 m thick ice, with occasional icebreaking.

### 2.1. Post-Processing—General Protocol and Principles

Conversion of the echosounder recordings to meaningful marine biological information first involves a rather coarse post-processing, which is usually performed onboard by scrutinizing echograms. Since the Institute of Marine Research (IMR) developed acoustic surveys for fish abundance estimation in the Nordic Seas in the sixties [20], a formal protocol for this process has been developed and adjusted according to available technology. At least once a day, the chief instrument operator and the cruise leader sit together in the acoustic centre of the research vessel and replay the echosounder recordings using the LSSS software (Version 2.16.0) [13,14]. For every unit nautical distance, usually five nautical miles (nmis), the recordings of volume back-scattering strength are converted to nautical area back-scattering coefficient (NASC, s_A_ with the unit (m^2^/(nmi)^2^). The recordings are usually separated in convenient depth layers, and there are possibilities to limit recordings of dense shoals or schools using a school function. NASC values are displayed for the different depth layers and school units and integrated for the unit nautical distance. Before allocation to biological categories like zooplankton and fish, the recordings are “cleaned” for unwanted back scattering from surface disturbance, for instance, air plumes in the water column during bad weather, false bottom, interference from other acoustic instruments, noise from different sources, or irregular bottom detections. The LSSS system has a convenient rubber function for erasing noisy recordings, and a region removal function for deleting nautical distances with many disturbances or distances where the ship has turned on acoustic registrations to trawl against the planned cruise direction. Surface and bottom irregularities are usually handled with redrawing the upper surface layer and the lower bottom layer manually using a pencil function. Also, the pencil function is often used to adjust the set depth layers to delimitate recordings that vary in depth extent. The final allocation of NASC for every unit nautical distance to given taxa or families of specific species is performed manually by setting the NASC value or the percentage of the total NASC value. In both cases, the total NAC value for the given unit nautical distance should add up to one hundred percent. With the LSSS system, it is possible to analyse echosounder recordings not only for nautical distance sailed, but also by pings or time as the basic units in cases when the echosounders are stationary, or geographical information is not connected to the echosounder recordings.

The main purpose of the post-processing procedure onboard is to “judge” or “scrutinize” the recordings so that the NASC origination from marine biological sources like zooplankton or fish can be allocated to taxonomic units such as order, families, or species. This can be performed based on the characteristics of the recordings, or through sampling of the back-scattering organisms with trawl or plankton nets. Thus, the post-processing onboard is somewhat subjective [21,22], and to complete it as objectively as possible, the IMR protocol states that it shall be performed by the chief instrument operator in cooperation with the cruise leader or an acoustic specialist. The chief instrument operator is the one operating the computer systems and is responsible for the storing of both the raw and processed data onboard, and for transferring the acoustic recordings and other logged data from the specific cruise to the onshore database at the IMR headquarter in Bergen.

To help allocate the recorded NASC to plankton or other scattering categories during the post-processing onboard, aimed pelagic trawling and plankton net surveys were undertaken (Figure 1). These surveys were performed in leads in ice-covered waters with an ice-rigged Harstad pelagic trawl [23] with an opening of about 250 m^2^ [18], and the MIK ring net (3.14 m^2^ opening, 1500 μm mesh size in the main net and 500 μm in the last 1 m) and MultiNet (1 m^2^ opening, 180 μm mesh size), plankton samplers hauled vertically. The trawl hauls were performed at about a 300 m depth over a distance of about 1.5 nautical miles (towed at 30 min at 3 knots).

### 2.2. Post-Processing of Echosounder Recordings from the Arctic Ocean

The echosounder recordings from the Arctic Ocean surveys in July–August 2022 and 2023 were manually scrutinized onboard according to the general protocol and principles outlined above. The recordings from the 38 kHz channel were chosen for post-processing, and with a minimum detection threshold of −82 dB as normally used during fisheries acoustic surveys. Aimed pelagic trawling was conducted to identify recordings on the Svalbard shelf and on the slope north of Svalbard with the conventional Harstad trawl and a larger Vito pelagic trawl (Figure 1). Based on the catches obtained, it was possible to identify single recordings, and to separate mixed recordings to taxa, species groups, and single fish species. Precise monitoring of the gear performance and the catching process gives vital information to support separation of the back-scattering organisms.

**Figure 2:**
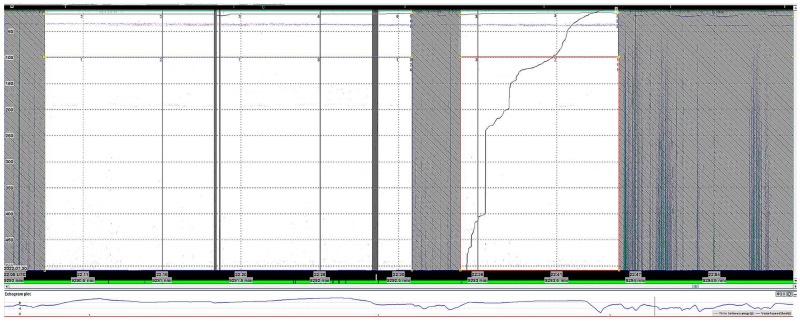
Scrutiny of the 38 kHz Simrad EK80 recordings using the LSSS post-processing software. Screen dump from the LSSS after post-processing of a five nautical mile distance along the 41° W longitude from the North Pole to the transect. Shaded areas removed because of noise, and 641 of 3320 pings used for final allocation of s_A_—values to the different species groups (here, plankton in the upper 100 m layer and mesopelagic/plankton in the deeper layer). The line rising from the bottom to the top in the white window marked by a red square to the right shows the relative, aggregated S_A_—value for that window. The lower window shows the speed (in knots) of the vessel.

During the data scrutiny onboard, noise in the recordings due to icebreaking, or other interactions with ice floes, were removed using the convenient region removal function of the LSSS system (Figure 2). For distances where heavy ice conditions were encountered, there were a few 5 nmi sections where the recordings had to be removed completely because of continuous noise recordings. Especially towards the end of the southward hydrographic transect in August 2022, after the Nansen-1 mooring deployment, we encountered large, dense ice floes which the ship had to break through. Nevertheless, for most 5 nmis sailed, there were sections with representative recordings, generally when the ship was navigated through open leads in ice-covered waters. Then, the allocation of area backscattering coefficients to the recording categories was possible

**Figure 3:**
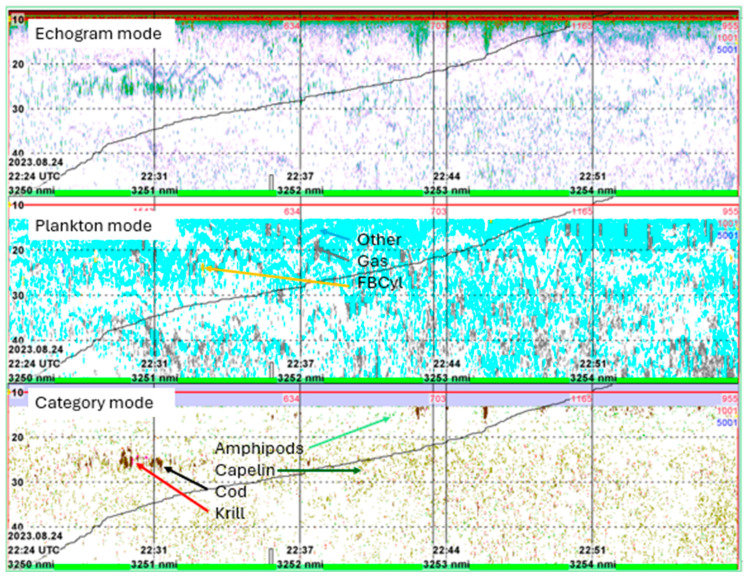
LSSS display of upper 60 m of a five nautical mile recording (log3250–3255) north of Pipps Island, Svalbard, 24 August 2023. Normal echogram displayed at −82 dB detection threshold (**upper**), in *Plankton mode* (**middle**) using the KORONA facility, and in *Category mode* (**lower**) using the KORONA facility. The Other, Gas, and FBCyl categories marked in the *Plankton mode* refer to the plankton model categories identified (further explanation in the text). The Amphipods, Capelin, Cod, and Krill in the *Category mode* refer to categories identified through the ALC library (further explanation in the text). The oblique line from bottom left to top right in all modes is the relative, aggregated s_A_—value for the 10–50 m depth channel for 5 nmi (log 3250–3255) integrated.

The echosounder recordings were scrutinized a second time at the IMR acoustic laboratory in Bergen aided by KORONA, a pre-processing facility of the LSSS, that carries out time-intensive processing of acoustic data [14]. The settings and results from the onboard scrutiny post-processing was the starting point for the second post-processing. These data were then replayed using the KORONA facility to remove impulse noise and ambient noise from the acoustic recordings, and thereafter also for the categorization of the acoustic recordings based on an acoustic feature-library incorporated into the LSSS (see [14] for further explanation of LSSS and the KORONA facility). To help distinguish fish with swim bladder from other scatterers, the *Plankton mode* of the KORONA suite was used regularly. This module is based on the multi-frequency backscatter of theoretical models of fluid-bent cylinder, prolate spheroid, hard-shelled, and gas-filled targets [24,25,26] incorporated into a framework for estimating zooplankton specimen size [2]. These models, in turn, fit well with backscatter from euphausiids or amphipods (fluid-bent cylinder, FBCyl), copepods (prolate spheroid), siphonophores, or small mesopelagic fish with small swim bladder (gas-filled targets, Gas), or hard-shelled zooplankton such as the sea butterfly (*Limacina helicina)*, or other organisms (Other, middle panel of Figure 3. In addition to the model-based targets, the content of the acoustic feature-library contained known recordings of fishes like capelin (*Mallotus villosus*), cod (*Gadus morhua*), euphausiids (krill, *Thyssanoessa* sp.), and siphonophores. For all five nautical miles sampled during the two cruises, the *Categorization mode* was used. This mode enables distinguishing recordings in several Acoustic Library Categories (ALCs): here, we applied amphipods, capelin, cod, or krill (lower panel of Figure 3). Each ALC is trained on a verified acoustic registration of that category, e.g., ALC_capelin includes the acoustic characteristics of capelin. Based on the visual results of the *Categorization mode* and the trawl samples obtained during the cruises, the following categories were applied for the allocation of NASC values: amphipods, capelin, cod, redfish, krill, mesopelagics (including some zooplankton and the glacier lanternfish [*Benthosema glacialis*]), O—group fish (for the 2023 recordings only), and plankton. Due to the weak recordings made by the Simrad EK80 echosounder in the CAO, the LSSS software was developed further to give the NASC (s_A_) with three decimals (normally only integers are used). The final allocation of NASC to taxa, order, families of specific species was performed manually.

During the second round of post-processing, the recording conditions when the ship was moving or lying still for station work (CTD and vertical net hauls) were especially considered. The LSSS recordings from just 19 of the undertaken 80 CTD stations were stored as part of the categorization of the integrated NASC. This was mostly due to the ship not moving during the CTD stations in densely ice-covered waters. When run in *Distance averaged mode*, LSSS does not store values if the vessel is stationary. On some occasions, the LSSS data from the CTD stations were excluded because there was too much noise during the CTD stations due to other acoustic instrument activity (use of ADCP or multibeam echo sounding).

## 3. Results

**Figure 4:**
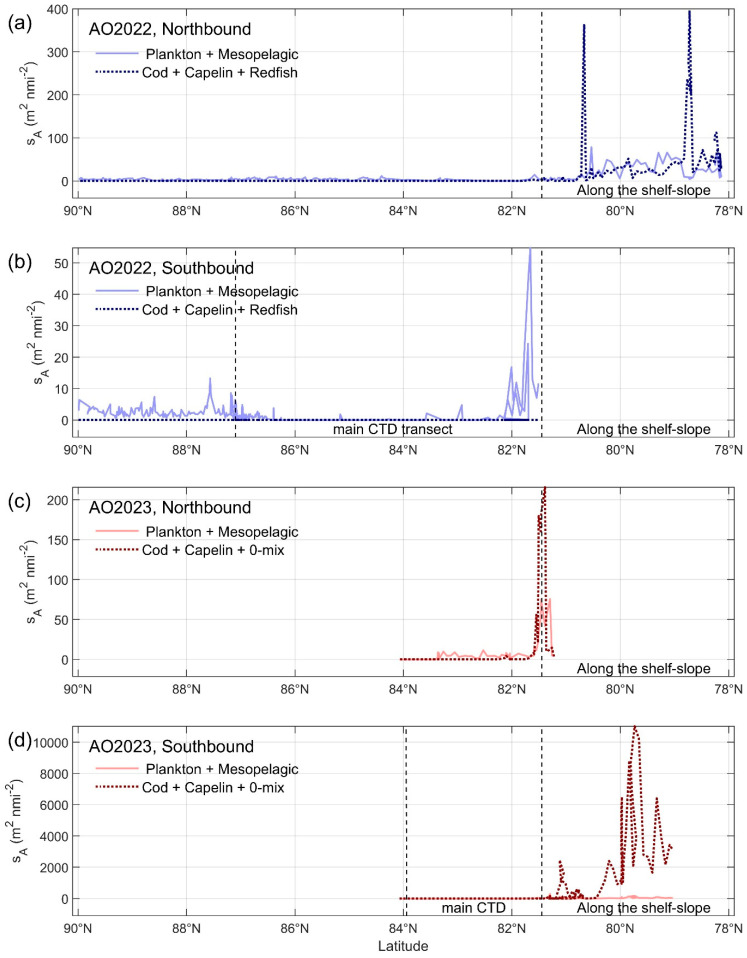
Recordings of cod (COD), capelin (CAP), redfish (RED), 0-group fish (0-MIX), mesopelagics (MEPLs), and plankton (PLANK) by the Simrad EK80 echosounder at 38 kHz and post-processed in LSSS during the Arctic Ocean cruise 2022 and 2023. (**a**) Northbound recordings in 2022, and (**b**) southbound in 2022. (**c**) Northbound recordings in 2023, and (**d**) southbound in 2023.

**Figure 5:**
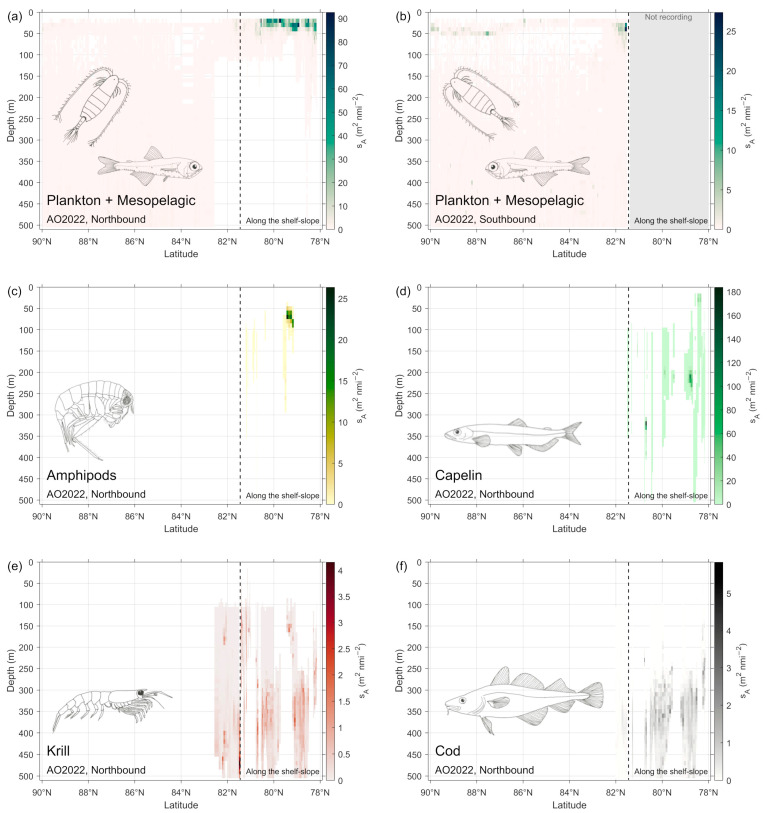
Recordings during the 2022 survey of (**a**,**b**) mesopelagics and plankton on the northbound and southbound part of the survey, respectively, and (**c**) amphipods, (**d**) capelin, (**e**) krill, and (**f**) cod on the northbound part of the survey.

The two step post-processing of the acoustic recordings using LSSS show substantial recordings of fish like capelin, cod, and redfish on the shelf west and north of Svalbard in July–August 2022 (Figure 4 and Figure 5). There were recordings of plankton near the surface on the shelf, and of a weak mesopelagic layer from the slope north of Svalbard and north to the North Pole (Figure 5). South from the North Pole there were some recordings of plankton and a weak mesopelagic layer. Similarly, the two step post-processing of the acoustic recordings from the shorter 2023 survey gave about the same picture with good recordings of fish and plankton on the Svalbard shelf and a weak mesopelagic layer and some plankton off the shelf north of Svalbard (Figure 4). In 2023, the area back scatter from fish on the shelf west and north of Svalbard on the way south was more than ten times the strength of the recordings in 2022. This was because there was a high abundance of young-of-the year fish (O—group) of capelin, cod, and other species in 2023.

## 4. Discussion

By use of a rather time consuming and quite labour intensive two step post-processing procedure, we have been able to provide quite coherent results of the distribution of plankton, fish, and mesopelagic organisms in the Arctic Ocean, from the Svalbard shelf to the North Pole. We have taken advantage of the state-of-the-art acoustic equipment and sampling devices that can be operated from R/V “*Kronprins Haakon*”, and through the LSSS system been able to delete noise from icebreaking and other disturbances. Through the KORONA module and the plankton mode of the LSSS system, and the information obtained from directed trawl and plankton net sampling, we have been able to convert the recordings to nautical area back-scattering strengths to biological taxa as plankton and fish, to plankton categories like amphipods and euphausiids, and even to fish species such as capelin, cod, and redfish.

The second post-processing gave added value in the conversion of the area back scattering to the biological units including taxa, families or specific species. This was facilitated by the KORONA facility which enabled separation of the area back scattering to crustacean families as amphipods and euphausiids, and likewise to fish species such as capelin and cod. The latter was also performed manually during the post-processing onboard, but with more certainty during the second post-processing based on the separation of the back-scattering objects on the LSSS display. Especially, when substantial recordings of young-of-the year fish were encountered on the way south on the shelf and along the western coast of Svalbard, the KORONA-based separation of the recordings was very useful. Often such recordings are difficult to separate during manual allocation of the area back-scattering values.

Based on our standardization and description, the overall results from the surveys would likely have been quite similar if the echo recordings had been scrutinized by different teams. However, differences might also have occurred because of substantial subjective elements in the scrutinizing process, in spite of a quite strict protocol for how the process should be carried out. Even if this is a well-known element in the marine acoustic survey process, there are few studies addressing the subjectivity in allocation of acoustic recordings to taxa, families, and species during marine acoustic surveys. Korsbrekke and Misund [21] found substantial heterogeneities in the judging of acoustic records between different teams. In a recent study, Fall et al. [22] found that seven teams scrutinizing the same data from Barents Sea acoustic and trawl surveys largely agreed on the total acoustic energy attributable to marine organisms, but there was significant variation in how the teams split this energy to different categories of marine organisms. This was mainly caused by differences in applied thresholds for separating fish and plankton recordings among the teams, as well as disagreements in species classification. Quite remarkably, the effect of the teams either dominated or was of a similar magnitude to the variability between segments when scaled up to the number of acoustic segments in a typical survey. Fall et al. [22] argued that further standardization of the scrutinizing process is needed to reduce the variability among different teams in allocating acoustic energy to different categories of marine organisms. Application of algorithms with elements of machine learning as the KORONA module in LSSS or the krill separation algorithm in Echoview [12] will help in standardizing the allocation of area back-scattering strengths to different categories of marine organisms.

Certain elements of machine learning are built into the KORONA module of LSSS enabling allocation to taxa, categories, and even species. In the future development of post-processing systems, such modules could be developed further based on modern principles of machine learning [27,28,29] and possibly also artificial intelligence. Using deep convolutional neural networks, sand eel (*Ammodytes* sp.) schools could quite reliably be distinguished from other fish schools and the sea bottom [30,31,32]. Instead of the quite laborious two step post-process presented here, future processing of acoustic recordings should be based on giving the initial boundary conditions of ocean area to be surveyed, depths to be anticipated for given geographic positions, and taxa, categories, and species of plankton and fish expected to be recorded. Biological information from aimed trawling and plankton nets could be entered underway. Then, the future post-processing system should be able to convert the volume back scatter recorded from the echosounder unit to nautical area back scattering for different taxa, categories, and species in the area, and finally to biomass of the taxa, categories, and fish species in the area surveyed. In future post-processing systems, user friendliness should be prioritized. Existing systems are quite user- and data capacity-demanding, with numerous options and settings to consider before starting to use the systems. For a basic survey, the use of a number of the settings and options should be reduced. Hopefully, such a development will result in systems that contribute to the better monitoring of marine ecosystems both in polar waters and other challenging areas.

As is evident from our study, much of the scrutinising process onboard was about removing sections with icebreaking-induced noise. The allocation of area back-scattering strength to taxa, families, and species was straightforward, however. In boreal waters with a higher diversity, the allocation to taxa, families, and species of marine organisms may be more complex. And in tropical waters with a regionally high diversity, it is necessary to group the allocation to just a few taxa and family groups like plankton, pelagic group I (often clupeoids, anchovies, sardines), a pelagic II group (carangids, mackerels, etc.), and a demersal group (groupers, etc.). With a development of post-processing systems as outlined above, hopefully the allocation of area back-scattering strengths to different marine organisms will be better resolved even in areas with a higher diversity. With an increasing pressure on the living marine resources of the world, such a development may help in advising on sustainable fisheries [33].

## Data Availability

The cruises from which the data analysed in this study are reported by Dodd et al. 2022 [17] and Hop et al. 2023 [19]. In these cruise reports, information is given about the storage of the data, and from where the data can be accessed.

## Abbreviations

The following abbreviations are used in this manuscript:

AO2022	Arctic Ocean cruise 2022
AO2023	Arctic Ocean cruise 2023
CAO	Central Arctic Ocean
IMR	Institute of Marine Research
KPH	*R/V Kronprins Haakon*
NASC	nautical area back-scattering strength, s_A_ with the unit *m*^2^*/(nautical mile)*^2^ = *m*^2^ *nmi^−^*^2^ [34]
NPI	The Norwegian Polar Institute
